# Simultaneous Measurement of Changes in Mitochondrial and Endoplasmic Reticulum Free Calcium in Pancreatic Beta Cells

**DOI:** 10.3390/bios13030382

**Published:** 2023-03-14

**Authors:** Sivakumar Jeyarajan, Irina X Zhang, Peter Arvan, Stephen I. Lentz, Leslie S. Satin

**Affiliations:** 1 Department of Pharmacology, University of Michigan Medical School, Ann Arbor, MI 48105, USA; jeyaraja@umich.edu (S.J.);; 2 Department of Internal Medicine, Division of Metabolism, Endocrinology & Diabetes, University of Michigan Medical School, Ann Arbor, MI 48105, USA; 3 Department of Molecular & Integrative Physiology, University of Michigan Medical School, Ann Arbor, MI 48105, USA

**Keywords:** genetically encoded calcium sensors, live cell imaging, insulin, metabolism, oscillations

## Abstract

The free calcium (Ca^2+^) levels in pancreatic beta cell organelles have been the subject of many recent investigations. Under pathophysiological conditions, disturbances in these pools have been linked to altered intracellular communication and cellular dysfunction. To facilitate studies of subcellular Ca^2+^ signaling in beta cells and, particularly, signaling between the endoplasmic reticulum (ER) and mitochondria, we designed a novel dual Ca^2+^ sensor which we termed DS-1. DS-1 encodes two stoichiometrically fluorescent proteins within a single plasmid, G-CEPIA-er, targeted to the ER and R-CEPIA3-mt, targeted to mitochondria. Our goal was to simultaneously measure the ER and mitochondrial Ca^2+^ in cells in real time. The *K_ds_* of G-CEPIA-er and R-CEPIA3-mt for Ca^2+^ are 672 and 3.7 μM, respectively. Confocal imaging of insulin-secreting INS-1 832/13 expressing DS-1 confirmed that the green and red fluorophores correctly colocalized with organelle-specific fluorescent markers as predicted. Further, we tested whether DS-1 exhibited the functional properties expected by challenging an INS-1 cell to glucose concentrations or drugs having well-documented effects on the ER and mitochondrial Ca^2+^ handling. The data obtained were consistent with those seen using other single organelle targeted probes. These results taken together suggest that DS-1 is a promising new approach for investigating Ca^2+^ signaling within multiple organelles of the cell.

## 1. Introduction

The Ca^2+^ levels of intracellular organelles are critical for maintaining proper cell function and cellular homeostasis [1,2,3]. These levels are regulated by the concerted interaction of the membrane ATPases that transport Ca^2+^ at the expense of ATP [4,5,6,7], as well as various Ca^2+^ binding proteins [8,9,10]. ER function has been of particular interest because of the important role of the ER in maintaining cytosolic Ca^2+^ levels and for the role of the ER Ca^2+^ in protein folding within the ER lumen [11,12]. In the mitochondria, which supplies cellular energy to the cell in the form of ATP, Ca^2+^ regulates pyruvate dehydrogenase, a Krebs cycle enzyme that helps regulate the available cellular energy supply [13,14,15,16].

Ca^2+^ communication between the ER and the mitochondria is mediated by the MAMS (Mitochondrial Associated Membranes) [17,18,19,20,21], functional contacts between the ER and the mitochondria that can transport Ca^2+^ and other signals between the two organelles. MAMs are composed of various proteins, including IP3R [22,23] or RyR [24,25] ER Ca^2+^ channels [26], VDAC [27], GRP75 [28], GRP78 [22] and MCU1 [29,30]. By mediating connections between these organelles, MAMs enable Ca^2+^ to move from the mitochondrion to the ER. The number and efficiency of MAM contact points have been shown to be modulated during different physiological states [31] or in response to disease processes, including diabetes [13,32,33,34].

To facilitate functional studies of the ER to mitochondrial Ca^2+^ communication and to better understand the role of MAMs in insulin-secreting pancreatic beta cells [2,28,35], we took advantage of a relatively new family of Ca^2+^ reporter molecules called CEPIAs [36] These reporters have been shown to be excellent probes for monitoring free Ca^2+^ in many different types of organelles [37,38,39]. Here we show that two different CEPIAs having distinct spectra and free Ca^2+^ affinities can be combined in a single plasmid to preserve probe stoichiometry to measure the ER and mitochondrial Ca^2+^ simultaneously. To accomplish this, we made a plasmid that contained the sequences of two different CEPIAs separated by an intervening T2A peptide sequence. This allowed the two CEPIA Ca^2+^ sensor sequences to be transcribed into a single mRNA that was, in turn, translated as two proteins due to ribosomal skipping [40]. Successful segregation of the two probes to their respective organelles allowed dual recordings of the ER and mitochondrial Ca^2+^ to be made simultaneously using live cell imaging in real time.

## 2. Materials and Methods

### 2.1. Cell Culture 

Rat insulinoma cells INS-1 832/13 cells were cultured in RPMI medium containing 11 mM glucose, 10% fetal bovine serum, 10 mM HEPES (N-2-hydroxyethylpiperazine-N′-2-ethanesulfonic acid), 1 mM sodium pyruvate and 55 µM beta-mercaptoethanol. Penicillin and streptomycin were added to reach a final concentration of 100 units/mL. 

### 2.2. Construction of the Dual Sensor

DS-1 was made by fusing two sensors, pCMV G-CEPIA1-er (Addgene plasmid # 58215; a gift from Dr. Masamitsu Iino) and R-CEPIA3-mt (a codon-optimized synthetic gene). Sequences were cloned in frame and separated by the self-cleaving peptide T2A. T2A was used to enable the expression of equimolar concentrations in each of the two sensors [40,41,42,43,44]. To accomplish this, G-CEPIA-er was restriction digested with *Not*I and *Xba*I. Subsequently, the genes for T2A and R-CEPIA3-mt were PCR amplified and cloned in frame with G-CEPIA-er using NEBuilder HiFi DNA assembly master mix (New England Biolabs, Ipswich, MA, USA). The construct was then authenticated by Sanger sequencing in the University of Michigan DNA Sequencing Core. The annotated DNA and amino acid sequences are shown in Supplementary Materials Figures S7 and S8.

### 2.3. DS-1 Expression Using Adenovirus 

To overcome the characteristic low efficiency of lipofectamine-mediated transfection, DS-1 was packaged in an adenovirus which was then used to infect INS-1 832/13 cells. For generating the requisite adenovirus, DS-1 was cloned in frame with a CMV promoter into a pACCMV2 adenovirus shuttle vector provided by the University of Michigan Vector Core. INS-1 832/13 cells (1 × 10^5^) were seeded onto glass-bottomed 35 mm tissue culture dishes (Fluorodishes; WPI, Sarasota, FL, USA) and cultured overnight. Cells were transduced the next day by incubating the cells for 3 h with AdV containing DS-1 at a viral titer of 0.003 × 10^9^ pfu/mL; the MOI as calculated was 30. Following incubation, the medium containing virus was removed and the cells were washed 2× with fresh medium. Cells were imaged 48 h post transduction. More than 90% of transduced INS-1 cells expressed both DS-1 fluorophores using this protocol. 

### 2.4. Solutions Used and Method of Solution Exchange during Live Cell Imaging

For free Ca^2+^ measurements, standard imaging buffer contained 140 mM NaCl, 3 mM CaCl_2_, 5 mM KCl, 2 mM MgCl_2_, 10 mM HEPES and 11 mM glucose. All solutions were made fresh on the day of the experiment by diluting frozen stocks. 

Imaging solutions were exchanged manually by pipette during confocal imaging by aspirating off one solution and dispensing the next. A reserve volume of 500 µL was maintained in each Fluoro-dish during these exchanges. To modulate the Ca^2+^ levels of the two respective organelles, cyclopiazonic acid (CPA; Cayman Chemicals, Ann Arbor, MI, USA), potassium chloride (KCl) or sodium azide (NaN_3_; Sigma Aldrich, St. Louis, MO, USA) was added to the experimental chamber in standard imaging buffer at 2× of their working concentrations. Similarly, drugs or chemicals were removed from the chamber by repeated exchanges with imaging solution. For the glucose stimulation experiments, imaging solution with and without glucose was applied to the cells.

### 2.5. Microscopy and Imaging

Live cell imaging was carried out using a Nikon A1 laser scanning confocal microscope equipped with NIS Elements software (Nikon Instruments Inc., Melville, NY, USA) to automate both microscope scanning and photomultiplier tube detectors. To compensate for possible drift during image acquisition, the Nikon Perfect Focus System was used. Cells were imaged with a 40×, 1.3 NA Nikon oil immersion objective at 37 °C within a TOKAI HIT environmental chamber (Tokai, Shizuoka, Japan).

To perform live cell Ca^2+^ imaging, INS-1 832/13 cells were transduced with DS-1 for most experiments but also transfected individually with G-CEPIA-er or R-CEPIA3-mt for control experiments. G-CEPIA-er excitation was provided by a 488 nm Argon laser and emission was collected at 500–530 nm, while R-CEPIA3-mt was excited by a 543 nm HeNe laser and emission was collected at 553–618 nm. Images were collected every 10 s. Acquired images were 1024 × 1024 pixels (318.51 µm), and image resolution was 0.31 µm/pixel. With the aforementioned settings, the rate of scanning required for each fluorophore was 2.25 s per frame.

### 2.6. Localization of DS-1 within the Cell

**Figure 1:**
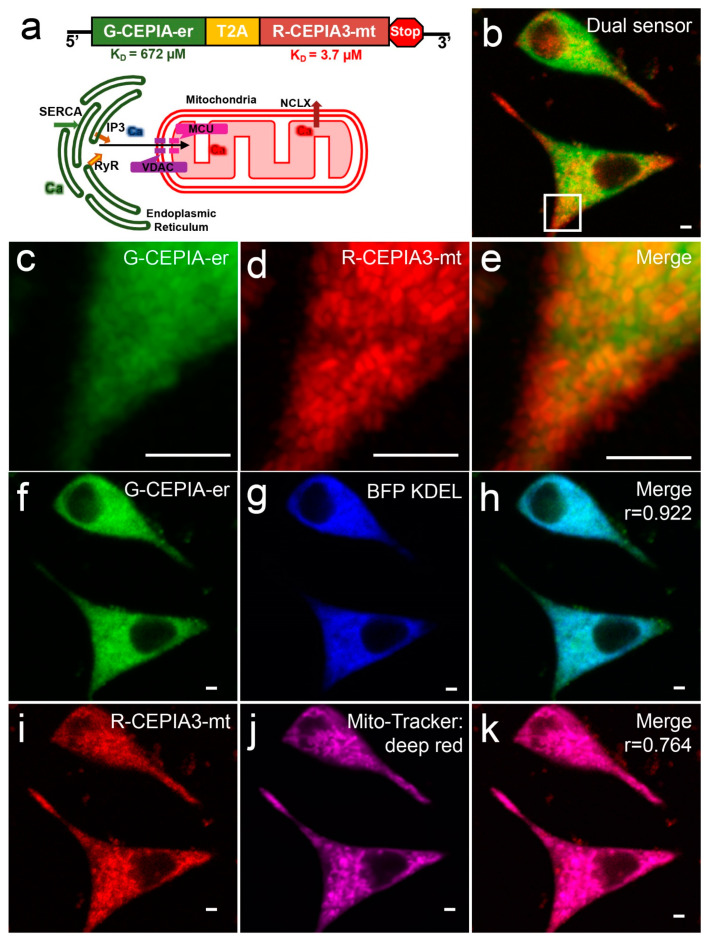
Colocalization of DS-1 to ER and mitochondria. (**a**) Schematic representation of DS-1 showing G-CEPIA-er fused to R-CEPIA3-mt with T2A self-cleaving peptide. The binding affinities of ER and mito Ca^2+^ sensors are shown below the schema. The exchange of Ca^2+^ within the organelles is shown in the cartoon. The fluorescence associated with Ca^2+^ binding to DS-1 in their respective organelles is indicated by their color cues. (**b**) Shows two INS-1 832/13 cells expressing DS-1. Confocal image of INS-1 832/13 cell transfected with DS-1 imaged with green and red laser lines. The enlarged insets shown depict the green (**c**), red (**d**) and a merger of both channels (**e**). The green channel of DS-1 (**f**) and the blue channel of the ER marker BFP-KDEL (**g**) are also indicated. Colocalization of G-CEPIA-er and BFP-KDEL are in (**h**). The Red channel of DS-1 (**i**) and the far-red channel of Mitotracker deep red (**j**). Co-localization of R-CEPIA3-mt paired with Mitotracker deep red is shown in (**k**). The correlation values of the merged colors were obtained from JaCOP plugin in ImageJ and their values are indicated. The scatter plots for the colocalized images shown in Figure 1b,h,k are shown in Figure S1a–c. The scale bar represents 2 µm.

After constructing DS-1 and expressing it in cells, the localization and function of the two distinct fluorophores of the plasmid were evaluated using confocal microscopy as shown in Figure 1. TagBFP-KDEL (Addgene plasmid # 49150) was chosen as an ER marker, and Mito-Tracker deep red (Thermo Scientific, Waltham, MA, USA) was selected to mark mitochondria. Excitation and emission wavelengths of the two markers were as follows: Tag BFP-KDEL:ER marker (Ex 405 nm and Em 425–475 nm) and Mito Tracker deep red (Ex 638 nm and Em 663–738 nm). The specific cell of interest was zoomed at 10× using the previously defined confocal settings to capture colocalization images. The correlation coefficients obtained for each of the respective markers and DS-1 were calculated using the JACoP Plugin [45] of ImageJ [46].

### 2.7. Analysis and Processing of Acquired Live Cell Imaging of Ca^2+^

**Figure 2:**
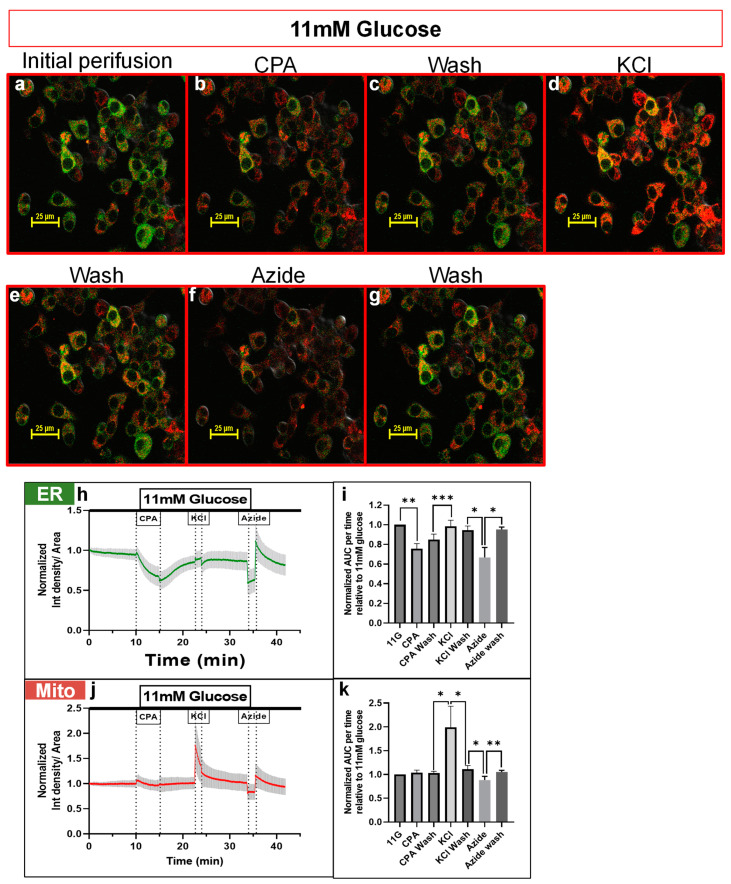
Functional characterization of DS-1 transduced INS1-832/13 cells with agents known to affect ER and mito Ca^2+^. Cells were initially treated with imaging buffer and exchanged with specific agents dissolved in imaging buffer. Live cell images were taken continuously every 10 s during the process. (**a**–**g**) Snapshot of confocal image for each treatment condition and washes. The complete time series is shown in Movie S1. (**h**,**j**) Mean ER and mito fluorescent value of all the cells in the field with their standard deviation. The arbitrary fluorescent value of each cell is calculated from ImageJ. (**i**,**k**) Quantitation of change in fluorescence represented as normalized AUC (area under curve) relative to 11 mM glucose (imaging buffer) for each treatment condition and their statistical summary. * *p* < 0.1; ** *p* < 0.05 and *** *p* < 0.01. Representative ER and mito traces of individual cells are shown in Figure S3.

**Figure 3:**
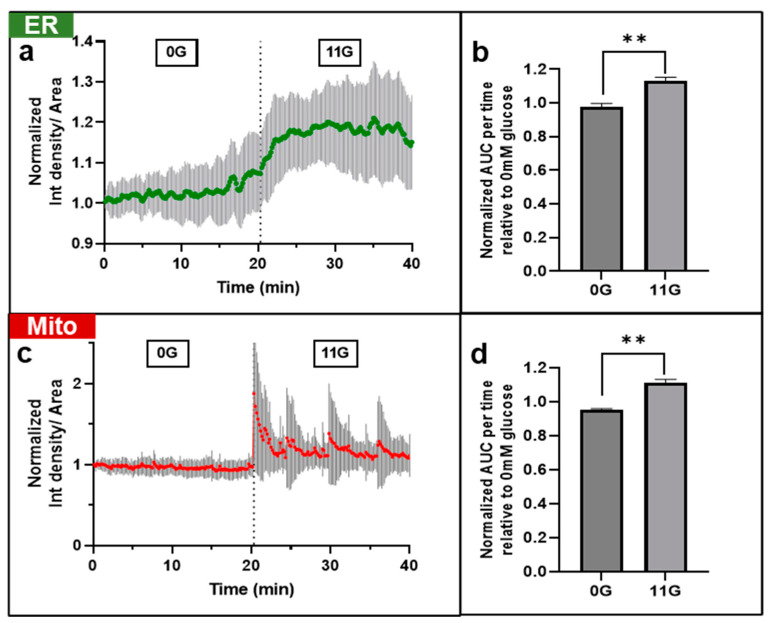
Characterization of the glucose responses in INS1-832/13 cells expressing DS-1. (**a**,**c**) Mean fluorescent traces of ER and mito with standard deviation in gray lines. (**b**,**d**) Quantitation of response illustrated as normalized AUC (area under curve) relative to 0 mM glucose (initial perifusion solution). ** represents statistical significance *p* < 0.05. Representative ER and mito traces of individual cells are shown in Figure S6.

After acquisition, images were saved as 8-bit stacked TIFF files and analyzed using ImageJ software as in [47]. Briefly, regions of interest (ROI) were drawn around individual cells, including the nucleus of the cell. As some cells exhibited movement in the scanning field at times while undergoing imaging, we drew ROIs that were larger than the individual cells to ensure that the cell images remained inside the ROIs despite small movements. Subsequently, ROIs were split into their respective red or green channels and a multi-measure prompt option was selected to obtain fluorescent intensity values within the selected cell regions. No masks were applied to fluorescent images. Values of integral density/area vs. time were plotted using GraphPad Prism (GraphPad, San Diego, CA, USA) [11]. Area under the curve (AUC) values for graphed data were calculated using Prism for each treatment condition. AUC values were then normalized and plotted over time. All time series data were plotted relative to their initial values. Data from at least 150 cells were analyzed and are reported in Figure 2. Data from at least 120 cells are reported in Figure 3.

### 2.8. Data Analysis and Statistics

Data were analyzed using GraphPad Prism. Standard error of the mean (S.E.M.) and Student’s *t*-test (two-tailed paired with criteria of significance: * *p* < 0.1; ** *p* < 0.05 and *** *p* < 0.01) were calculated.

## 3. Results

### 3.1. DS-1 Correctly Localized CEPIA-er and CEPIA-mito to Their Targeted Organelles

Figure 1a shows the structure of DS-1 whereby the two respective CEPIA probes, G-CEPIA-er and R-CEPIA-mt, were separated by a T2A sequence. The location of the 5′ and 3′ ends of the construct are indicated, as well as the placement of a stop codon at the 3′ end. A cartoon shown beneath depicts schematically the location in the cell of the Ca^2+^ components SERCA, IP3 and RyR channels, MCU and NCLX.

To determine whether each sensor was correctly localized to its intended organelle when DS-1 was expressed, we used the blue fluorescent protein ER marker BFP-KDEL [48] and the far-red marker Mitotracker, as in [49]. The green and red channels corresponding to G-CEPIA-er and R-CEPIA-mt are shown together in INS1-832/13 cells in Figure 1b. In these cells, both green (ER) and red (mitochondrial) areas can be seen. Figure 1c,d display an enlarged section of the image for the green and red channels. A merger of both channels is displayed in Figure 1e, showing separation of the red and green regions with no overlap between them. This indicates that the ER and mito probes are localized to individual organelles. An additional image supporting this is shown in Figure S1d. 

In Figure 1f,i, individual channels showing red vs. green emission are shown, along with either blue-fluorescent protein (BFP) linked to KDEL to mark the ER (Figure 1g) or Mitotracker Red to mark mitochondria (Figure 1j). The merged images and their correlation coefficients, which were determined from the images in the merged panel, are displayed in Figure 1h,k in the far-right columns. Visual inspection combined with the semiquantitative analysis of the confocal images confirmed that the green ER Ca^2+^ sensor, G-CEPIA-er, and the ER marker, BFP-KDEL, were colocalized, yielding a correlation coefficient of 0.922. The green and blue signals observed were excluded from the nucleus of the cell and appeared to take on a cytoplasmically distributed pattern consistent with that of the ER [36]. Additionally, the red mitochondrial Ca^2+^ sensor, R-CEPIA3-mt, colocalized with the mitochondrial standard (correlation coefficient of 0.764). The red- and magenta-colored organelles showed a Mitotracker pattern with areas of staining showing small spherical- or oval-shaped tubules. Both the morphology we observed and the colocalization of the two CEPIAs with the standard organelle markers strongly indicated that the sensors were correctly localized to the organelles of interest. 

### 3.2. DS-1 Expressing Cells Responded Appropriately to Drugs Targeting ER or Mitochondrial Ca^2+^ Pools

When observed using confocal microscopy and standard imaging buffer with 11 mM of glucose, INS1-832/13 cells exhibited their characteristic oval-spheroid shape, with some elongated extensions apparent in individual cells. The cells clearly contained red and green fluorescence, although the patterns and intensities of the green and red signals within the cells were heterogenous (Figure 2a). While some individual cells showed more green fluorescence than red, in others, red fluorescence was prominent, or both colors were visible, including regions of red/green overlap, visible as patches of yellow. In these, signals from the ER and the mitochondrial Ca^2+^ pools appeared to overlap strongly. The variability we observed in the subcellular fluorescence could reflect differences in the respective trafficking of the two CEPIAs to their respective target organelles or could reflect differences in the Ca^2+^ concentrations of the corresponding mitochondrial or ER Ca^2+^ pools. 

To monitor functional changes in the ER and mitochondrial Ca^2+^, live cell imaging was performed. Images were collected every 10 s, and cells were exposed to an imaging buffer (as above) and then the imaging buffer, which contained agents that have been previously shown to alter either the ER or mitochondrial Ca^2+^. The image stack acquired is shown as Movie S1 in the Supplementary Materials. Still images taken of a representative field of cells corresponding to each treatment condition are shown in Figure 2a–g. The treatments tested are listed at the top of each panel. The time courses of individual G-CEPIA-er and R-CEPIA3-mt traces are shown in Figures S4 and S5.

The mean (+/−sd) time course of the ER Ca^2+^ that was observed in response to various treatments carried out over more than 40 min is shown in Figure 2h, while the corresponding time course for the mitochondrial Ca^2+^ collected simultaneously is shown in Figure 2j. Analysis of changes in the ER and mitochondrial signals (as normalized AUC, see Methods) is shown in Figure 2i,k, respectively. The statistical summary was obtained from three independent experiments, and asterisks denote a significance level at * *p* < 0.1; ** *p* < 0.05 and *** *p* < 0.01.

Following an initial period in the control solution exposure to 25 μM CPA, a reversible inhibitor of ER SERCA activity [50] promptly reduced the ER Ca^2+^, as evidenced by a marked reduction in the level of green fluorescence while red fluorescence attributed to mitochondrial Ca^2+^ was still clearly visible (Figure 2b). CPA removal resulted in increased green fluorescence, consistent with the restoration of the ER Ca^2+^, as pumping was resumed (Figure 2c). Interestingly, the decrease in green fluorescence seen upon CPA addition corresponded to an enhanced level of red fluorescence as well (Figure 2b). Quantification of the fluorescence values obtained in Figure 2j confirmed there was a transient increase in red fluorescence that was restored to its basal level within a few minutes.

To determine whether raising the cytosolic Ca^2+^ in turn affected the ER and mitochondrial Ca^2+^, we added 30 mM KCl to the INS1-832/13 cells to depolarize them to open the voltage-gated Ca^2+^ channels (VDAC) [51]. As shown in Figure 2d,h–k, membrane depolarization caused a modest increase in ER Ca^2+^ but a much larger increase in mitochondrial Ca^2+^. Washing KCl reduced the Ca^2+^ levels of each organelle pool (Figure 2e).

As the Ca^2+^ pumping and K(ATP) channel closure are both metabolically regulated processes in beta cells [52,53], we added sodium azide to the cells. Azide lowers ATP/ADP in the cytosol by inhibiting complex III (cytochrome oxidase) of the mitochondrial electron transport chain [54]. We thus predicted that azide would block the mitochondrial Ca^2+^ uptake and reduce the ER Ca^2+^ by lowering ATP/ADP. As can be seen in Figure 2f–k, azide addition promptly reduced green and red fluorescence, consistent with an action to lower both the ER and mitochondrial Ca^2+^ levels. After removing the azide, both red and green fluorescence levels were recovered (Figure 2g).

### 3.3. DS-1 Fluorescence Responded to Changes in Glucose Concentration

To test how DS-1 and its respective G-CEPIA-er and R-CEPIA-mt signals responded to changes in glucose, INS-1 832/13 cells expressing DS-1 were bathed in glucose-free imaging solution and were then exposed to a solution containing 11 mM of glucose. Raising glucose concentration causes a rise in cytosolic Ca^2+^ due to the K(ATP) channel closure [55]. Increasing the glucose concentration resulted in a sustained rise in the ER and mitochondrial Ca^2+^, as shown in Figure 3a,c. The integrated responses of DS-1 to glucose were statistically significant, as shown in Figure 3b,d for both the ER and mitochondrial Ca^2+^ (obtained from three independent experiments). Specifically, INS-1 cells exhibited either single free Ca^2+^ transients or sustained oscillations atop a small plateau. Traces of a few individual cells showing these heterogenous responses are shown in Figure S6. However, only 20–30% of our cells showed consistent responses to rises in glucose, likely because of the heterogenous nature of INS1-832/13 cells [56,57,58,59,60], which can exhibit quite variable responses to glucose. We also noted that in some INS1-832/13 cells, the mitochondria exhibited robust Ca^2+^ oscillations, as can be seen in Figure 3c’s lower panel. These have been reported by other investigators using different approaches to measure mitochondrial Ca^2+^ [61,62,63]. In our study, mitochondrial oscillation was typically observed following a pronounced rise in ER Ca^2+^.

## 4. Discussion

We herein describe a novel dual Ca^2+^ sensor that we designed by fusing two genetically engineered Ca^2+^ probes, one targeted specifically to the ER (G-CEPIA-er) [36] and the other to the mitochondria (R-CEPIA3-mt) [64]. Using this new tool allowed us to measure Ca^2+^ simultaneously in both organelles and within the same cells. The gene construct was transduced into insulin-secreting INS1-832/13 cells using standard adenovirus-based methodology. We validated that DS-1 and its resultant ER and mitochondrial Ca^2+^ reporters were correctly targeted both by morphological criteria and by colocalization with standard organellar markers.

It has been shown previously that two or three genetically encoded Ca^2+^ indicators (GECIs) [36,65,66] can be expressed in the same cell using co-transfections [67,68] or sequential transductions [69,70,71,72,73]. This allows two different Ca^2+^ sensors to be expressed within different organelles, similar to our current study. However, the chance that each target cell receives the same ratio of the two genetically engineered genes is statistically low; selecting cells expressing probes with similar ratios in a mixed population is time-consuming and requires that certain assumptions are made. In contrast, an advantage of our single plasmid approach is that it ensures that the stoichiometry of the two different Ca^2+^ probes is fixed and both are expressed in the same cell. Additionally, multiple rounds of sequential transduction are avoided in our approach. DS-1 was developed to enable us to simultaneously study two organellar Ca^2+^ pools within the same cell and with the same stoichiometry after a single transduction. By targeting specific cellular compartments, DS-1 also avoids the limitations inherent to small fluorescent dyes, such as fura-2, which only report the Ca^2+^ levels of the cytosol and which cannot be targeted to the interior of specific organelles.

The functional properties of DS-1 were validated using pharmacological agents that target specific organellar Ca^2+^ pools. As expected, the ER Ca^2+^ decreased following CPA exposure and increased following CPA removal. KCl-mediated plasma membrane depolarization caused a modest increase in the ER Ca^2+^ but a large increase in the mitochondrial Ca^2+^. Briefly treating the cells with azide decreased both the ER and mitochondrial Ca^2+^, which returned to their initial levels after the azide removal. It is known that azide action is reversible [74,75].

We also used DS-1-expressing INS-832/13 cells to test the glucose responsiveness of the ER and mitochondria. Glucose enters the beta cell, and its metabolism subsequently leads to the closure of the K(ATP) channels [76]. This activates the voltage-dependent Ca^2+^ channels to increase cytosolic Ca^2+^ [51]. This, in turn, results in an increase in both the ER and mitochondrial Ca^2+^ (Figure 3a,c) [77]. This finding is generally consistent with earlier reports of the ER Ca^2+^ studied by use of the FRET probe, D4ER and mitochondrial Ca^2+^ measurements made using mitopericam [62,78], mitochondrially targeted aequorin [61,79,80] or other probes [81].

To the best of our knowledge, this is the first report showing that the ER and mitochondrial free Ca^2+^ can be measured simultaneously with a single plasmid in pancreatic beta cells. The approach is promising, in our view, because the overall strategy we employed to make DS-1 can be used to make other dual or even triple Ca^2+^ probes or probes for other metabolites of interest such as ATP [82], which are targeted to various organelles. The endless varieties of possibilities are exciting and may well lead to many new discoveries.

An obvious limitation of CEPIA-based constructs is, that being intensitometric probes, they only report relative signal levels rather than ratios. Ratiometric probes can be more sensitive to changes in levels of the analyte and may be less susceptible to changes in the optical path length or variations in probe concentration [83,84]. In addition, ratiometric probes can be more readily calibrated to absolute levels of the species being detected. We emphasize that the general dual-sensor/multi-sensor method developed for this study can be extended to ratiometric probes, as well as to study a variety of cell types.

## 5. Conclusions

In summary, we have developed a novel means to simultaneously monitor the Ca^2+^ levels of distinct intracellular compartments in pancreatic beta cells. Future work will incorporate additional compartments and different fluorophores for real-time imaging of metabolism in beta cells and other cell types.

## Data Availability

Data will be provided upon request.

## Supplementary Materials

The following supporting information can be downloaded at: https://www.zenodo.org/record/7735311#.ZBExWXZBxEY, Movie S1. Image series of DS-1-transduced INS1-832/13 cells treated by agents known to modulate the ER and mitochondria Ca^2+^. Green represents the ER Ca^2+^, and red represents the mitochondria Ca^2+^. The caption in the movie shows the treatment conditions. The movie shows the images taken once every 10 s; Figure S1: The figure (a–c) shows correlations between BFP-KDEL, MitoTracker, or G-CEPIA versus G-CEPIA, R-CEPIA and R-CEPIA, respectively; r values are Pearson’s correlation coefficients. Images shown at the bottom (in d) show green and red signals were nonoverlapping; Figure S2: To confirm that that the green and red fluorescent signals from G-CEPIA-er and R-CEPIA3-mt do not overlap, images acquired separately using argon (green) and neon (red) lasers are shown here. All images were acquired from cells recorded in standard imaging solution containing 11 mM glucose. DS-1 yielded signals in both the green and red channels, while G-CEPIA-er and R-CEPIA3-mt were only visible on their respective channels; Figure S3: Representative time courses of ER and Mito signals recorded from individual cells shown in Figure 2. Figure S4: Time courses of G-CEPIA-er traces where only the ER probe was transfected into cells; Figure S5: Time courses of R-CEPIA3-mt traces where only the Mito probe was transfected into cells; Figure S6: Representative ER and Mito traces shown for individual cells from Figure 3; Figure S7: (a) Plasmid map of DS-1. (b) Translated protein sequence for the DNA coding DS-1; Figure S8: Annotated sequence of DS-1 for (a) DNA and (b) amino acids. The G-CEPIA-er is shown in green and codon optimized R-CEPIA3-mt is shown in red. The T2A sequence is highlighted in yellow.

## Abbreviations

CEPIA—Calcium-Measuring Organelle-Entrapped Protein Indicator; SERCA—Sarco/Endoplasmic Reticulum Ca^2+^—ATPase; ER—Endoplasmic Reticulum; Mito—Mitochondria; IP3—Inositol-3-Phosphate; RyR—Ryanodine Receptor; CPA—cyclopiazonic acid; KCl—Potassium Chloride; Azide—Sodium Azide; AUC—area under curve; 0G—0 mM glucose; 11G—11 mM glucose.

## References

[1] Arruda A.P., Hotamisligil G.S. (2015). Calcium Homeostasis and Organelle Function in the Pathogenesis of Obesity and Diabetes. Cell Metab..

[2] Dingreville F., Panthu B., Thivolet C., Ducreux S., Gouriou Y., Pesenti S., Chauvin M.A., Chikh K., Errazuriz-Cerda E., van Coppenolle F. (2019). Differential Effect of Glucose on ER-Mitochondria Ca^2+^ Exchange Participates in Insulin Secretion and Glucotoxicity-Mediated Dysfunction of β-Cells. Diabetes.

[3] Rutter G.A., Tsuboi T., Ravier M.A. (2006). Ca^2+^ Microdomains and the Control of Insulin Secretion. Cell Calcium..

[4] Mourad N.I., Nenquin M., Henquin J.-C. (2010). Metabolic Amplifying Pathway Increases Both Phases of Insulin Secretion Independently of Beta-Cell Actin Microfilaments. Am. J. Physiol. Cell Physiol..

[5] Ravier M.A., Nenquin M., Miki T., Seino S., Henquin J.-C. (2009). Glucose Controls Cytosolic Ca^2+^ and Insulin Secretion in Mouse Islets Lacking Adenosine Triphosphate-Sensitive K+ Channels Owing to a Knockout of the Pore-Forming Subunit Kir6.2. Endocrinology.

[6] Johnson J.S., Kono T., Tong X., Yamamoto W.R., Zarain-Herzberg A., Merrins M.J., Satin L.S., Gilon P., Evans-Molina C. (2014). Pancreatic and Duodenal Homeobox Protein 1 (Pdx-1) Maintains Endoplasmic Reticulum Calcium Levels through Transcriptional Regulation of Sarco-Endoplasmic Reticulum Calcium ATPase 2b (SERCA2b) in the Islet β Cell. J. Biol. Chem..

[7] Beauvois M.C., Merezak C., Jonas J.-C., Ravier M.A., Henquin J.-C., Gilon P. (2006). Glucose-Induced Mixed [Ca^2+^]_c_ Oscillations in Mouse Beta-Cells Are Controlled by the Membrane Potential and the SERCA3 Ca^2+^-ATPase of the Endoplasmic Reticulum. Am. J. Physiol. Cell Physiol..

[8] Coe H., Michalak M. (2009). Calcium Binding Chaperones of the Endoplasmic Reticulum. Gen. Physiol. Biophys..

[9] Prins D., Michalak M. (2011). Organellar Calcium Buffers. Cold Spring Harb. Perspect. Biol..

[10] Venkatesan A., Satin L.S., Raghavan M. (2021). Roles of Calreticulin in Protein Folding, Immunity, Calcium Signaling and Cell Transformation. Prog. Mol. Subcell. Biol..

[11] Zhang I.X., Raghavan M., Satin L.S. (2020). The Endoplasmic Reticulum and Calcium Homeostasis in Pancreatic Beta Cells. Endocrinology.

[12] Hetz C., Zhang K., Kaufman R.J. (2020). Mechanisms, Regulation and Functions of the Unfolded Protein Response. Nat. Rev. Mol. Cell Biol..

[13] Tanaka T., Nagashima K., Inagaki N., Kioka H., Takashima S., Fukuoka H., Noji H., Kakizuka A., Imamura H. (2014). Glucose-Stimulated Single Pancreatic Islets Sustain Increased Cytosolic ATP Levels during Initial Ca^2+^ Influx and Subsequent Ca^2+^ Oscillations. J. Biol. Chem..

[14] Wacquier B., Combettes L., Dupont G. (2019). Cytoplasmic and Mitochondrial Calcium Signaling: A Two-Way Relationship. Cold Spring Harb. Perspect. Biol..

[15] Srinivasan M., Choi C.S., Ghoshal P., Pliss L., Pandya J.D., Hill D., Cline G., Patel M.S. (2010). SS-Cell-Specific Pyruvate Dehydrogenase Deficiency Impairs Glucose-Stimulated Insulin Secretion. Am. J. Physiol. Endocrinol. Metab..

[16] Denton R.M. (2009). Regulation of Mitochondrial Dehydrogenases by Calcium Ions. Biochim. Biophys. Acta.

[17] Patergnani S., Suski J.M., Agnoletto C., Bononi A., Bonora M., de Marchi E., Giorgi C., Marchi S., Missiroli S., Poletti F. (2011). Calcium Signaling around Mitochondria Associated Membranes (MAMs). Cell Commun. Signal..

[18] Joseph S.K., Booth D.M., Young M.P., Hajnóczky G. (2019). Redox Regulation of ER and Mitochondrial Ca^2+^ Signaling in Cell Survival and Death. Cell Calcium.

[19] Csordás G., Weaver D., Hajnóczky G. (2018). Endoplasmic Reticulum-Mitochondrial Contactology: Structure and Signaling Functions. Trends Cell Biol..

[20] Bagur R., Hajnóczky G. (2017). Intracellular Ca^2+^ Sensing: Its Role in Calcium Homeostasis and Signaling. Mol. Cell.

[21] Hayashi T., Rizzuto R., Hajnoczky G., Su T.-P. (2009). MAM: More than Just a Housekeeper. Trends Cell Biol..

[22] Csordás G., Várnai P., Golenár T., Roy S., Purkins G., Schneider T.G., Balla T., Hajnóczky G. (2010). Imaging Interorganelle Contacts and Local Calcium Dynamics at the ER-Mitochondrial Interface. Mol. Cell.

[23] Joseph S.K., Hajnóczky G. (2007). IP3 Receptors in Cell Survival and Apoptosis: Ca^2+^ Release and Beyond. Apoptosis.

[24] Roy Chowdhury A., Srinivasan S., Csordás G., Hajnóczky G., Avadhani N.G. (2020). Dysregulation of RyR Calcium Channel Causes the Onset of Mitochondrial Retrograde Signaling. iScience.

[25] Yi M., Weaver D., Eisner V., Várnai P., Hunyady L., Ma J., Csordás G., Hajnóczky G. (2012). Switch from ER-Mitochondrial to SR-Mitochondrial Calcium Coupling during Muscle Differentiation. Cell Calcium.

[26] García-Pérez C., Hajnóczky G., Csordás G. (2008). Physical Coupling Supports the Local Ca2+ Transfer between Sarcoplasmic Reticulum Subdomains and the Mitochondria in Heart Muscle. J. Biol. Chem..

[27] Zhang E., Mohammed Al-Amily I., Mohammed S., Luan C., Asplund O., Ahmed M., Ye Y., Ben-Hail D., Soni A., Vishnu N. (2019). Preserving Insulin Secretion in Diabetes by Inhibiting VDAC1 Overexpression and Surface Translocation in β Cells. Cell Metab..

[28] Tiwary S., Nandwani A., Khan R., Datta M. (2021). GRP75 Mediates Endoplasmic Reticulum-Mitochondria Coupling during Palmitate-Induced Pancreatic β-Cell Apoptosis. J. Biol. Chem..

[29] Hajnóczky G., Booth D., Csordás G., Debattisti V., Golenár T., Naghdi S., Niknejad N., Paillard M., Seifert E.L., Weaver D. (2014). Reliance of ER-Mitochondrial Calcium Signaling on Mitochondrial EF-Hand Ca^2+^ Binding Proteins: Miros, MICUs, LETM1 and Solute Carriers. Curr. Opin. Cell Biol..

[30] Berezhnaya E., Hajnóczky G. (2021). How Do MICUs Gate the Mitochondrial Calcium Uniporter?. Cell Calcium.

[31] Twig G., Liu X., Liesa M., Wikstrom J.D., Molina A.J.A., Las G., Yaniv G., Hajnóczky G., Shirihai O.S. (2010). Biophysical Properties of Mitochondrial Fusion Events in Pancreatic Beta-Cells and Cardiac Cells Unravel Potential Control Mechanisms of Its Selectivity. Am. J. Physiol. Cell Physiol..

[32] Sebastián D., Hernández-Alvarez M.I., Segalés J., Sorianello E., Muñoz J.P., Sala D., Waget A., Liesa M., Paz J.C., Gopalacharyulu P. (2012). Mitofusin 2 (Mfn2) Links Mitochondrial and Endoplasmic Reticulum Function with Insulin Signaling and Is Essential for Normal Glucose Homeostasis. Proc. Natl. Acad. Sci. USA.

[33] Cosson P., Marchetti A., Ravazzola M., Orci L. (2012). Mitofusin-2 Independent Juxtaposition of Endoplasmic Reticulum and Mitochondria: An Ultrastructural Study. PLoS ONE.

[34] Rieusset J. (2017). Role of Endoplasmic Reticulum-Mitochondria Communication in Type 2 Diabetes. Adv. Exp. Med. Biol..

[35] Madec A.M., Perrier J., Panthu B., Dingreville F. (2021). Role of Mitochondria-Associated Endoplasmic Reticulum Membrane (MAMs) Interactions and Calcium Exchange in the Development of Type 2 Diabetes. Int. Rev. Cell Mol. Biol..

[36] Suzuki J., Kanemaru K., Ishii K., Ohkura M., Okubo Y., Iino M. (2014). Imaging Intraorganellar Ca2+ at Subcellular Resolution Using CEPIA. Nat. Commun..

[37] Chang-Graham A.L., Perry J.L., Strtak A.C., Ramachandran N.K., Criglar J.M., Philip A.A., Patton J.T., Estes M.K., Hyser J.M. (2019). Rotavirus Calcium Dysregulation Manifests as Dynamic Calcium Signaling in the Cytoplasm and Endoplasmic Reticulum. Sci. Rep..

[38] Zhang L., Dietsche F., Seitaj B., Rojas-Charry L., Latchman N., Tomar D., Wüst R.C., Nickel A., Frauenknecht K.B., Schoser B. (2022). TMBIM5 Loss of Function Alters Mitochondrial Matrix Ion Homeostasis and Causes a Skeletal Myopathy. Life Sci. Alliance.

[39] Korecka J.A., Talbot S., Osborn T.M., de Leeuw S.M., Levy S.A., Ferrari E.J., Moskites A., Atkinson E., Jodelka F.M., Hinrich A.J. (2019). Neurite Collapse and Altered ER Ca2+ Control in Human Parkinson Disease Patient IPSC-Derived Neurons with LRRK2 G2019S Mutation. Stem Cell Rep..

[40] Liu Z., Chen O., Wall J.B.J., Zheng M., Zhou Y., Wang L., Vaseghi H.R., Qian L., Liu J. (2017). Systematic Comparison of 2A Peptides for Cloning Multi-Genes in a Polycistronic Vector. Sci. Rep..

[41] Schwirz J., Yan Y., Franta Z., Schetelig M.F. (2020). Bicistronic Expression and Differential Localization of Proteins in Insect Cells and Drosophila Suzukii Using Picornaviral 2A Peptides. Insect Biochem. Mol. Biol..

[42] Platisa J., Vasan G., Yang A., Pieribone V.A. (2017). Directed Evolution of Key Residues in Fluorescent Protein Inverses the Polarity of Voltage Sensitivity in the Genetically Encoded Indicator ArcLight. ACS Chem. Neurosci..

[43] Niopek D., Wehler P., Roensch J., Eils R., Di Ventura B. (2016). Optogenetic control of nuclear protein export. Nat. Commun..

[44] Daniels R.W., Rossano A., Macleod G., Ganetzky B. (2014). Expression of Multiple Transgenes from a Single Construct Using Viral 2A Peptides in Drosophila. PLoS ONE.

[45] Bolte S., Cordelières F.P. (2006). A guided tour into subcellular colocalization analysis in light microscopy. J. Microsc..

[46] Schneider C.A., Rasband W.S., Eliceiri K.W. (2012). NIH Image to ImageJ: 25 Years of image analysis. Nat. Methods.

[47] Sun Z., Südhof T.C. (2020). A simple Ca^2+^-imaging approach to neural network analyses in cultured neurons. J. Neurosci. Methods.

[48] Friedman J.R., Lackner L.L., West M., DiBenedetto J.R., Nunnari J., Voeltz G.K. (2011). ER Tubules Mark Sites of Mitochondrial Division. Science.

[49] Zhou R., Yazdi A.S., Menu P., Tschopp J. (2011). A role for mitochondria in NLRP3 inflammasome activation. Nature.

[50] Seidler N.W., Jona I., Vegh M., Martonosi A. (1989). Cyclopiazonic Acid is a Specific Inhibitor of the Ca^2+^-ATPase of Sarcoplasmic Reticulum. J. Biol. Chem..

[51] Merrins M.J., Poudel C., McKenna J.P., Ha J., Sherman A., Bertram R., Satin L.S. (2016). Phase Analysis of Metabolic Oscillations and Membrane Potential in Pancreatic Islet β -Cells. Biophys. J..

[52] Thompson B., Satin L.S. (2021). Beta-Cell Ion Channels and Their Role in Regulating Insulin Secretion. Compr. Physiol..

[53] Bertram R., Satin L.S., Sherman A.S. (2017). Closing in on the Mechanisms of Pulsatile Insulin Secretion. Diabetes.

[54] Misler S., Falke L.C., Gillis K., McDaniel M.L. (1986). A metabolite-regulated potassium channel in rat pancreatic B cells. Proc. Natl. Acad. Sci. USA.

[55] Islam M.S. (2010). Calcium Signaling in the Islets. Adv. Exp. Med. Biol..

[56] Merglen A., Theander S., Rubi B., Chaffard G., Wollheim C.B., Maechler P. (2004). Glucose Sensitivity and Metabolism-Secretion Coupling Studied during Two-Year Continuous Culture in INS-1E Insulinoma Cells. Endocrinology.

[57] Skelin M. (2010). Pancreatic beta cell lines and their applications in diabetes mellitus research. Altex.

[58] Miyazaki S., Tashiro F., Tsuchiya T., Sasaki K., Miyazaki J.-I. (2021). Establishment of a long-term stable β-cell line and its application to analyze the effect of Gcg expression on insulin secretion. Sci. Rep..

[59] Goehring I., Gerencser A.A., Schmidt S., Brand M.D., Mulder H., Nicholls D.G. (2012). Plasma Membrane Potential Oscillations in Insulin Secreting Ins-1 832/13 Cells Do Not Require Glycolysis and Are Not Initiated by Fluctuations in Mitochondrial Bioenergetics. J. Biol. Chem..

[60] Neal A.S., Rountree A.M., Radtke J.R., Yin J., Schwartz M.W., Hampe C.S., Posner J.D., Cirulli V., Sweet I.R. (2016). A method for high-throughput functional imaging of single cells within heterogeneous cell preparations. Sci. Rep..

[61] Kennedy E.D., Rizzuto R., Theler J.M., Pralong W.F., Bastianutto C., Pozzan T., Wollheim C.B. (1996). Glucose-stimulated insulin secretion correlates with changes in mitochondrial and cytosolic Ca^2+^ in aequorin-expressing INS-1 cells. J. Clin. Investig..

[62] Tarasov A.I., Semplici F., Ravier M.A., Bellomo E.A., Pullen T.J., Gilon P., Sekler I., Rizzuto R., Rutter G.A. (2012). The Mitochondrial Ca^2+^ Uniporter MCU Is Essential for Glucose-Induced ATP Increases in Pancreatic β-Cells. PLoS ONE.

[63] Montemurro C., Nomoto H., Pei L., Parekh V.S., Vongbunyong K.E., Vadrevu S., Gurlo T., Butler A.E., Subramaniam R., Ritou E. (2019). IAPP toxicity activates HIF1α/PFKFB3 signaling delaying β-cell loss at the expense of β-cell function. Nat. Commun..

[64] Kanemaru K., Suzuki J., Taiko I., Iino M. (2020). Red fluorescent CEPIA indicators for visualization of Ca^2+^ dynamics in mitochondria. Sci. Rep..

[65] Hirabayashi Y., Kwon S.-K., Paek H., Pernice W.M., Paul M.A., Lee J., Erfani P., Raczkowski A., Petrey D.S., Pon L.A. (2017). ER-mitochondria tethering by PDZD8 regulates Ca^2+^ dynamics in mammalian neurons. Science.

[66] Arai S., Kriszt R., Harada K., Looi L.-S., Matsuda S., Wongso D., Suo S., Ishiura S., Tseng Y.-H., Raghunath M. (2018). RGB-Color Intensiometric Indicators to Visualize Spatiotemporal Dynamics of ATP in Single Cells. Angew. Chem. Int. Ed..

[67] Sun C., Shui B., Zhao W., Liu H., Li W., Lee J.C., Doran R., Lee F.K., Sun T., Shen Q.S. (2019). Central role of IP3R2-mediated Ca^2+^ oscillation in self-renewal of liver cancer stem cells elucidated by high-signal ER sensor. Cell Death Dis..

[68] Su S., Phua S.C., DeRose R., Chiba S., Narita K., Kalugin P.N., Katada T., Kontani K., Takeda S., Inoue T. (2013). Genetically encoded calcium indicator illuminates calcium dynamics in primary cilia. Nat. Methods.

[69] Tian L., Hires S.A., Mao T., Huber D., Chiappe M.E., Chalasani S.H., Petreanu L., Akerboom J., McKinney S.A., Schreiter E.R. (2009). Imaging neural activity in worms, flies and mice with improved GCaMP calcium indicators. Nat. Methods.

[70] Okubo Y., Iino M. (2019). Visualization of astrocytic intracellular Ca^2+^ mobilization. J. Physiol..

[71] Kempmann A., Gensch T., Offenhäusser A., Tihaa I., Maybeck V., Balfanz S., Baumann A. (2022). The Functional Characterization of GCaMP3.0 Variants Specifically Targeted to Subcellular Domains. Int. J. Mol. Sci..

[72] Vargas M.E., Yamagishi Y., Tessier-Lavigne M., Sagasti A. (2015). Live Imaging of Calcium Dynamics during Axon Degeneration Reveals Two Functionally Distinct Phases of Calcium Influx. J. Neurosci..

[73] Vella S.A., Calixto A., Asady B., Li Z.-H., Moreno S.N.J. (2019). Genetic Indicators for Calcium Signaling Studies in Toxoplasma gondii. Methods Mol. Biol..

[74] Misler S., Barnett D.W., Falke L.C. (1992). Effects of metabolic inhibition by sodium azide on stimulus-secretion coupling in B cells of human islets of Langerhans. Pflug. Arch..

[75] Köhler S., Schmidt H., Fülle P., Hirrlinger J., Winkler U. (2020). A Dual Nanosensor Approach to Determine the Cytosolic Concentration of ATP in Astrocytes. Front. Cell. Neurosci..

[76] Satin L.S., Butler P.C., Ha J., Sherman A.S. (2015). Pulsatile insulin secretion, impaired glucose tolerance and type 2 diabetes. Mol. Asp. Med..

[77] Ravier M.A., Daro D., Roma L.P., Jonas J.-C., Cheng-Xue R., Schuit F.C., Gilon P. (2011). Mechanisms of Control of the Free Ca^2+^ Concentration in the Endoplasmic Reticulum of Mouse Pancreatic β-Cells. Diabetes.

[78] Nagai T., Sawano A., Park E.S., Miyawaki A. (2001). Circularly permuted green fluorescent proteins engineered to sense Ca^2+^. Proc. Natl. Acad. Sci. USA.

[79] Bermont F., Hermant A., Benninga R., Chabert C., Jacot G., Santo-Domingo J., Kraus M.R.-C., Feige J.N., De Marchi U. (2020). Targeting Mitochondrial Calcium Uptake with the Natural Flavonol Kaempferol, to Promote Metabolism/Secretion Coupling in Pancreatic β-cells. Nutrients.

[80] Rutter G.A., Theler J.M., Murgia M., Wollheim C.B., Pozzan T., Rizzuto R. (1993). Stimulated Ca^2+^ Influx Raises Mitochondrial Free Ca^2+^ to Supramicromolar Levels in a Pancreatic Beta-Cell Line. Possible Role in Glucose and Agonist-Induced Insulin Secretion. J. Biol. Chem..

[81] Suzuki J., Kanemaru K., Iino M. (2016). Genetically Encoded Fluorescent Indicators for Organellar Calcium Imaging. Biophys. J..

[82] Yong J., Bischof H., Burgstaller S., Siirin M., Murphy A., Malli R., Kaufman R.J. (2019). Mitochondria supply ATP to the ER through a mechanism antagonized by cytosolic Ca^2+^. Elife.

[83] Lee M.H., Kim J.S., Sessler J.L. (2015). Small molecule-based ratiometric fluorescence probes for cations, anions, and biomolecules. Chem. Soc. Rev..

[84] Bootman M.D., Rietdorf K., Collins T., Walker S., Sanderson M. (2013). Ca^2+^-Sensitive Fluorescent Dyes and Intracellular Ca^2+^ Imaging. Cold Spring Harb. Protoc..

